# Quantitative assessment of thenar to evaluate hand function after stroke by Bayes discriminant

**DOI:** 10.1186/s12891-023-06789-w

**Published:** 2023-08-29

**Authors:** Rui Li, Shuai Zheng, Yukang Zhang, Hongxia Zhang, Lijuan Du, Linggang Cheng, Hui Li, Wenkai Zhang, Kai Du, Wen He, Wei Zhang

**Affiliations:** 1 Department of Ultrasound, Beijing Tiantan Hospital, Capital Medical University, No. 119, South Fourth Ring Road West, Fengtai District, Beijing, 100070 China; 2 Department of Ultrasound, Baoji City Hospital of Traditional Chinese Medicine, Baoji, China

**Keywords:** Hand, Stroke, Bayes theorem, Rehabilitation

## Abstract

**Background:**

The incidence rate of stroke or cerebrovascular accidents ranks first in China. More than 85% of stroke patients have residual upper limb motor dysfunction, especially hand dysfunction. Normalizing the rehabilitation evaluation process and standard quantitative evaluation method is a complex and key point in rehabilitation therapy. The study aimed to establish a function model based on the Bayes discriminant by measuring the thenar stiffness with shear wave elastography (SWE) to quantitatively evaluate the hand motor function of hemiplegic patients after stroke.

**Methods:**

This study collected 60 patients diagnosed with hemiplegia after stroke from October 2021 to October 2022. Therapists used the Brunnstrom assessment (BA)scale to divide the patients into the stage. All the patients underwent the measurement of SWE examination of abductor pollicis brevis (APB), opponens pollicis (OP), flexor pollicis long tendon (FPLT), and flexor pollicis brevis (FPB) by two sonographers. The SWE change rate of four parts of the thenar area was calculated prospectively with the non-hemiplegic side as the reference, the function equation was established by the Bayes discriminant method, and the evaluation model was fitted according to the acquired training set data. Lastly, the model was verified by self-validation, cross-validation, and external data validation methods. The classification performance was evaluated regarding the area under the ROC curve (AUC), sensitivity, and specificity.

**Results:**

The median SWE values of the hemiplegic side of patients were lower than those of the non-hemiplegic side. According to the BA stage and SWE_R_ of APB, OP, FPLT, and FPB, our study established the Bayes discriminative model and validated it via self-validation and cross-validation methods. Then, the discriminant equation was used to validate 18 patients prospectively, the diagnostic coincidence rate was about 78.8%, and the misjudgment rate was approximately 21.2%. The AUC of the discriminant model for diagnosing BA stage I-VI was 0.928(95% CI: 0.839-1.0),0.858(95% CI: 0.748–0.969),1.0(95% CI: 1.0–1.0), 0.777(95% CI: 0.599–0.954),0.785(95% CI: 0.593–0.977) and 0.985(95% CI: 0.959-1.0), respectively.

**Conclusion:**

This Bayes discriminant model built by measuring thenar stiffness was of diagnostic value and can provide an objective basis for evaluating clinical rehabilitation.

## Introduction

Stroke or cerebrovascular accident is the second leading cause of death and disability in the world [1], whose incidence ranks first in China, with an increase of more than 2.5 million new cases yearly. According to statistics, more than 85% of stroke patients have leftover upper limb motor dysfunction [2, 3]. The living ability of patients after a stroke is associated with the recovery degree of upper limb motor function. Hands are involved in the most complex and elaborate activities, whose function accounts for about 90% of the upper limb function. The rehabilitation of hand function after stroke refers to using various evaluation methods for hand function rehabilitation to deliver clinical rehabilitation strategies and lower the disability rate of disease. Thus, rehabilitation evaluation is the foundation of rehabilitation therapy. Normalizing the rehabilitation evaluation process and standard quantitative evaluation method is a complex and key point in rehabilitation therapy.

Hand motor function is evaluated in clinical settings by clinical scales and tests [4]. BA scale is the most used hand function evaluation scale for stroke patients. Nevertheless, the BA scale is graded by rehabilitation therapists according to the therapist’s own experience. So traditional evaluation methods have a high degree of subjectivity, relying on the personal experience and subjective judgment of physicians. An objective and quantitative evaluation method is urgently needed in clinical practice [5]. In recent years, ultrasound shear wave elastography (SWE) has become a hot spot in the research of musculoskeletal diseases. It can be used to evaluate the changes in muscle stiffness and is of great clinical value for limb evaluation after stroke [6, 7]. This study attempted to provide a method for quantitatively evaluating hand function in patients with hemiplegia in clinical practice. SWE was used to measure the hardness of the thenar eminence of the patient’s hand in resting position, and the Bayes discriminant method was used to establish functional equations to match the training set data and BA scale, which verified the practicability of the data set evaluation model, and more objective data were used to evaluate the degree of rehabilitation of hand function of stroke patients.

## Materials and methods

### Patients

**Fig. 1:**
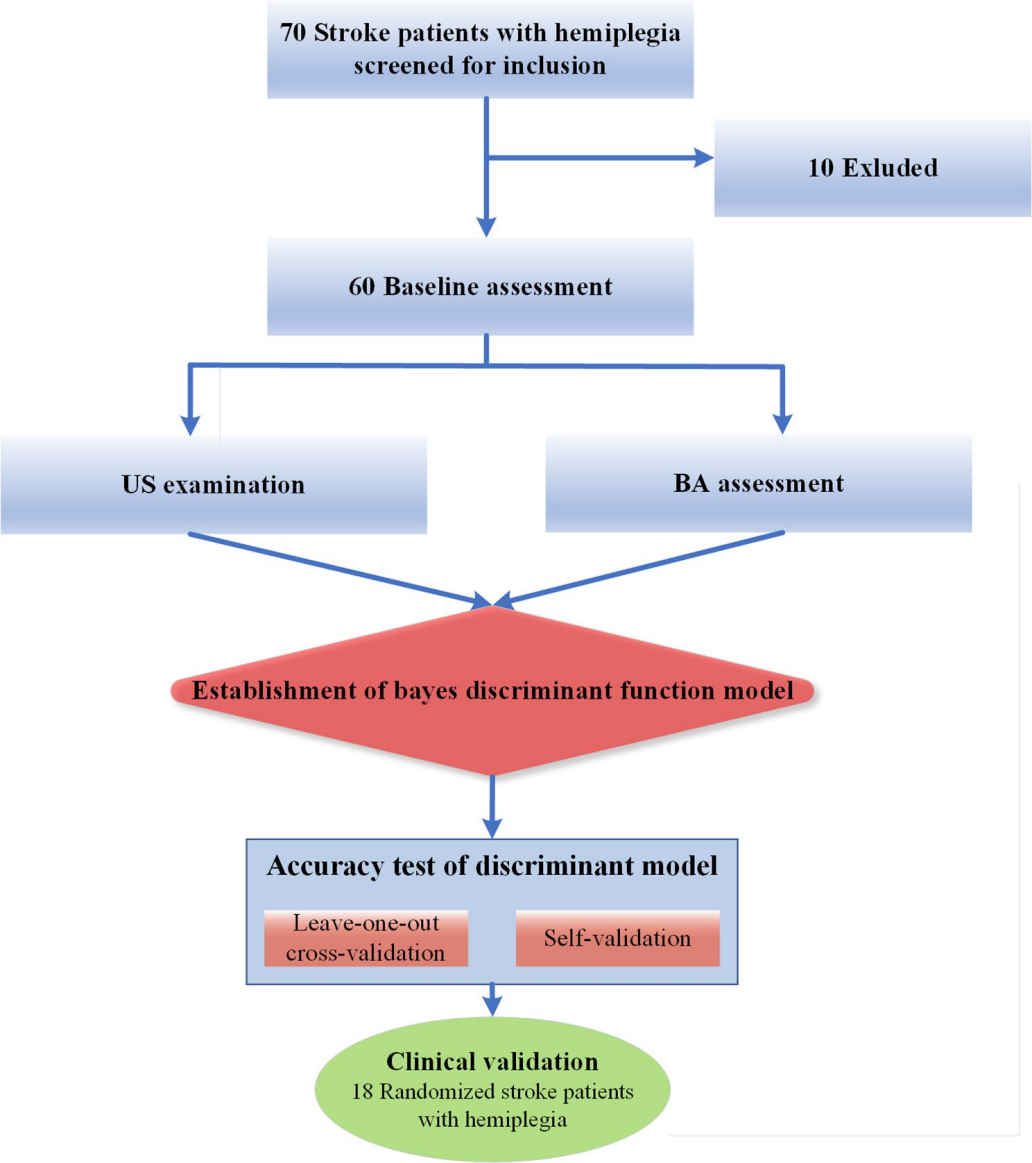
Flow Chart of This Study **Note**: Values are the number of patients.

In this cross-sectional and observational study, outpatients, and inpatients with hemiplegia for stroke presenting to Beijing Tiantan Hospital from October 2021 to October 2022 were consecutively enrolled. Inclusion criteria are patients diagnosed by imaging as having stroke accompanied by the first clinical manifestation of hand dysfunction (clinically determined); Conscious and able to cooperate with ultrasound examination, didn’t take anti-spasmodic drugs or a topical medicine. Exclusion criteria: (1) with unsteady vital signs; unconscious; (2) unable to cooperate in the examination; (3) with bilateral limb paralysis; (4) previous history of upper limb fracture; (5) muscle injury, brachial plexus injury, or surgery (Fig. 1). This prospective study had been approved by the Ethics Review Committee of Beijing Tiantan Hospital and was conducted by the Declaration of Helsinki (KYSQ 2019-039-01). All subjects signed informed consent before the test. Before the experiment, all patients underwent clinical physical examination, and two experienced rehabilitation therapists performed BA staging as the criterion to assess hand function.

### Ultrasonic examination

**Fig. 2:**
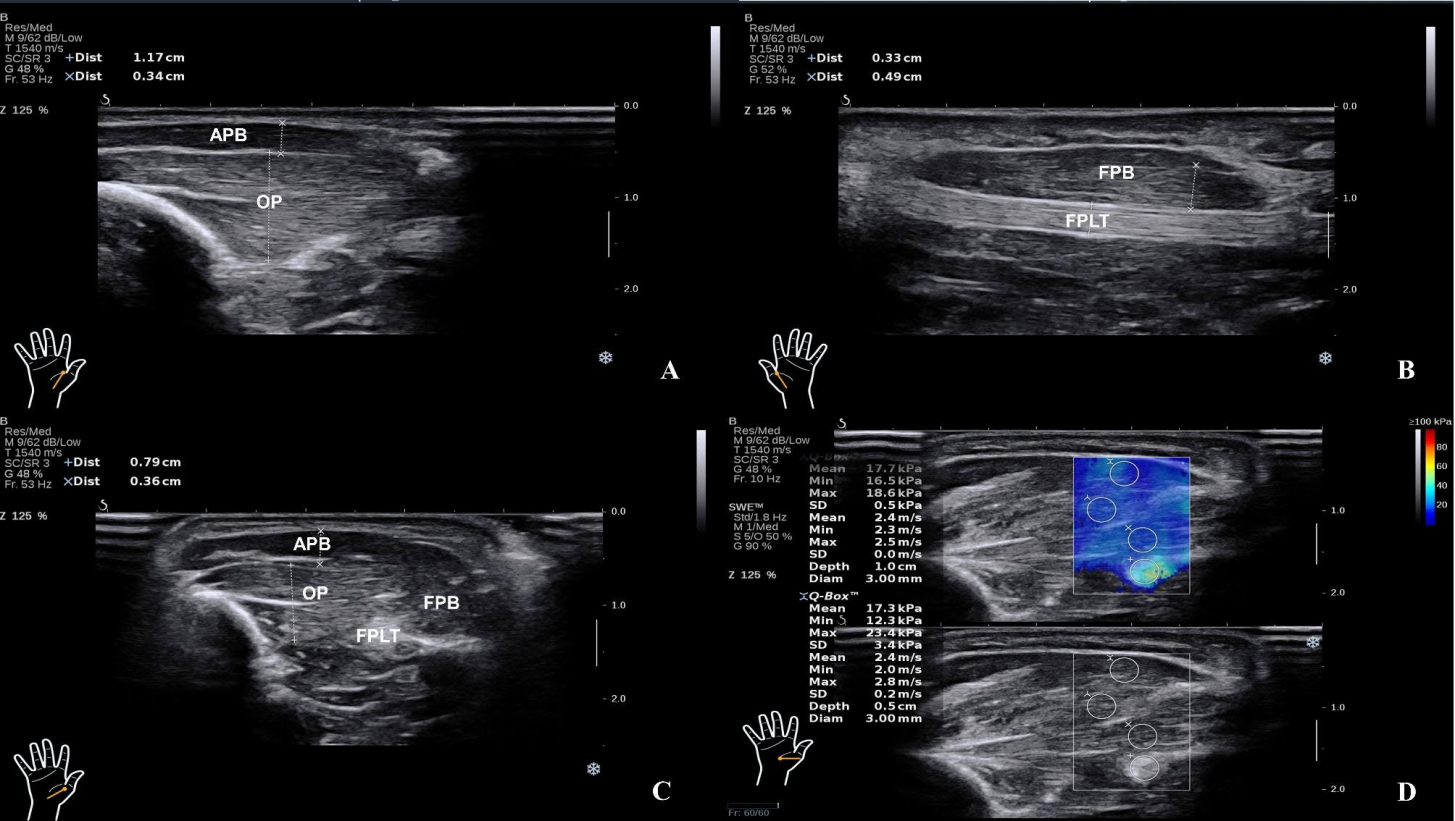
Schematic Diagram of Thenar Elasticity Measurement by SWE **Notes**: **A**: Transverse section shows opponens pollicis (OP) and abductor pollicis brevis (APB); **B**: Transverse section shows flexor pollicis brevis (FPB) and flexor pollicis long tendon (FPLT); **C**: Longitudinal section shows abductor pollicis brevis (APB), opponens pollicis (OP), flexor pollicis long tendon (FPLT), and flexor pollicis brevis (FPB); **D**: Longitudinal section shows SWE measurement of thenar muscles and tendons.

The SWE of APB, OP, FPLT, and FPB of major thenars of the hemiplegic hands and healthy hands of all of the patients included in this study were assessed by using an ultrafast US scanner (Aixplorer, Supersonic Imagine, France) driving a linear transducer array (SL 10 − 2, Supersonic Imagine). The muscle stiffness was measured in kilopascal (KPa), and the color code of Young’s modulus was set to 0-100 KPa. A sonographer in the process with 8–10 years of experience in musculoskeletal ultrasonography and elastography. The sonographer was unaware of the patient’s BA stage. During the ultrasonic evaluation, the room temperature was kept at 25 ± 2℃, and the subjects took supine and prone positions with their palms in an upward and naturally flexed state. This way, their hands can be stabilized, and the motion strain at the thenar eminence can be minimized. The first metacarpus and flexor pollicis longus tendon were taken as markers of the thenar eminence. To begin with, the longitudinal axis and short axis were combined to identify each structure, adjusted to SWE mode, and obtained color tissue elastogram. For the short axis of major thenar, the region of interest was placed in the muscle area to be measured. When the signals became stable, the image was frozen. APB, FPB, OP and FPLT were captured inside the region of interest and measured, with the diameter being set to 3 mm. The system automatically calculated the maximum, minimum, and mean Young’s modulus in the target area (Fig. 2). Three separate measurements were taken for each limb in a single position, and the mean Young’s modulus was calculated. During the measurement of SWE, the coupling agent was applied to the thenar thickly, the probe was immersed into the coupling agent, and a minimum probe pressure was applied. The SWE of the muscles and tendons of each subject was measured three times and then averaged. According to our previous studies, there was no significant difference between the left and right upper limbs of normal people in SWE values [8].

To rule out the effect of individual differences in muscle elasticity on analysis, we calculated the change rate (SWE_R_) of elasticity of major thenar on the hemiplegic side (SWEh) with the major thenar SWE (SWEn) on the non-hemiplegic side as the reference. The change rates of elasticity of APB, FPB, OP, and FPLT were denoted as APB-SWE_R_, FPB- SWE_R_, OP-SWE_R,_ and FPLT-SWE_R_ respectively.

$${SWE}_{R}=\frac{SWEn-SWEh}{SWEn}\times 100\%$$

### Clinical evaluation of hand function

The BA scale divided the rehabilitation process of stroke patients into six stages, according to the degree of spasm, synergy, and voluntary movement [9]:

Stage I Flaccidity is present, and no movements of the limbs can be initiated.

Stage II The basic limb synergies or some of their components may appear as associated reactions or minimal voluntary movement responses may be present. Spasticity begins to develop.

Stage III The patient gains voluntary control of the movement synergies, although the full range of all synergy components does not necessarily develop. Spasticity is severe.

Stage IV Some movement combinations that do not follow the synergies are mastered, and spasticity begins to decline.

Stage V More difficult movement combinations are possible as the elemental limb synergies lose dominance over motor acts.

Stage VI Spasticity disappears, and individual joint movements become possible.

### Statistical analysis

The data were analyzed by SPSS 28.0 statistical software. The Kolmogorov-Smirnov test was conducted to describe the normal distribution of all measured data; for example, age was expressed as mean ± SD, and one-way ANOVA was applied to analyze. SWE and other data were not normally distributed and described by median (lower quartile and upper quartile) [M (P25, P75)]. The differences between patient groups in gender and clinical etiology were compared using the χ2 test. Mann-Whitney U test was used to compare the SWE values of APB, FPB, OP, and FPLT on the hemiplegic and non-hemiplegic side of patients. The Kruskal-Wallis test was performed for multiple comparisons of BMI, and SWE_R_ among groups. By taking 60 patients as the experimental group, the SWE_R_ of APB, FPB, OP, and FPLT as independent variables, and the BA scale as the dependent variable, a Bayes discriminant equation was established and examined by self-validation and cross-validation to test the discriminant equation. Lastly, prospectively, 18 cases were pre-judged, and external data were validated for the discriminant equation. Receiver operating characteristic (ROC) curves were used to assess the diagnostic value of the Bayes discriminant function model in diagnosing the BA stage of patients’ hand function, and the area under the ROC curve (AUC) was calculated for each curve. The test level was α = 0.05. It was a two-tailed test, *P* < 0.05, indicating that the difference was statistically significant.

## Results

### Clinical baseline data

In this work, a total of 78 patients diagnosed with hemiplegia for stroke were included. All of them completed SWE measurements for thenars on the hemiplegic side and non-hemiplegic side, as well as limb function assessment.

As the experimental group of training samples, 60 patients with hemiplegia, including 39 males (65%), 21 females (35%), 17 cases (28.33%) of cerebral hemorrhage, and 43 cases (71.67%) of cerebral infarction were analyzed prospectively in this study. The mean age was 54.37 years. Among them, 34 patients (56.67%) had their left limb involved, and 26 patients had their right limb involved (43.33%), with an average course of 15.95 days. The BA stage of patients’ hand function: 19 cases (31.67%) in stage I, 10 cases (16.67%) in stage II, 7 cases (11.67%) in stage III, 10 cases (16.67%) in stage IV, 7 cases (16.67%) in stage V, and 7 cases (11.67%) in stage VI, and there was no significant difference between the six groups in age, sex, BMI, involve limbs (Table 1).

**Table 1:** Clinical data of the patient in Bruunstrom stage

Characteristics	Total	Bruunstrom Stage						*P*
		I	II	III	IV	V	VI	
Total	60(100%)	19(31.67%)	10(16.67%)	7(11.67%)	10(16.67%)	7(11.67%)	7(11.76%)	/
Gender^a^	60(100%)							> 0.05
Male	39(65%)	7(17.9%)	8(20.5%)	6(15.4%)	7(17.9%)	7(17.9%)	4(10.3%)	
Female	21(35%)	12(57.1%)	2(9.5%)	1(4.8%)	3(14.3%)	0(0.0%)	3(14.3%)	
Age^b^	54.37 ± 14.85	54.16 ± 14.21	47.30 ± 15.08	43.43 ± 17.45	58.70 ± 8.49	60.71 ± 5.88	63.43 ± 19.40	> 0.05
BMI^c^	25.55 (24.04,29.16)	27.68 (24.41,30.08)	25.21 (23.49,29.69)	26.67 (23.66,32.65)	24.72 (24.03,26.22)	26.44 (24.49,53.48)	24.97 (19.90,27.64)	> 0.05
Involved Limbs^a^	60(100%)							> 0.05
Left	34(56.67%)	6(17.6%)	8(23.5%)	5(14.7%)	9(26.5%)	1(2.9%)	5(14.7%)	
Right Side	26(43.33%)	13(50.0%)	2(7.7%)	2(7.7%)	1(3.8%)	6(23.1%)	2(7.7%)	
Clinical Diagnosis^a^	60(100%)							> 0.05
Encephalorrhagia	17(28.33%)	7(41.2%)	5(29.4%)	4(23.5%)	1(5.9%)	0(0.0%)	0(0.0%)	
Cerebral Infarction	43(71.67%)	12(27.9%)	5(11.6%)	3(7.0%)	9(20.9%)	7(16.3%)	7(16.3%)	

**Notes**: Values are the number of patients. a, Variable reported as the number of patients (percentage). Chi-Square Test was used to compare the differences between the six groups; b, Variable reported as mean ± standard deviation. One-way ANOVA was used to analyze the comparison between the two groups; the significance level is 0.05; c, Variable reported as the median (inter-quartile range, IQR), Kruskal-Wallis test for comparison between six groups, the significance level is 0.05.

As the test group of external data validation samples, 18 patients with hemiplegia were prospectively evaluated in this study, including 14 males (77.78%), 4 females (22.22%); 4 cases (22.22%) of cerebral hemorrhage, and 14 cases (77.78%) of cerebral infarction were analyzed prospectively in this study. The average age was 59.78 years. Among them, 10 patients (55.56%) had their left limb involved, and 8 patients had their right limb involved (44.44%). The BA stage of patients’ hand function: 4 cases (22.22%) in stage I, 5 cases (27.78%) in stage II, 0 case (0%) in stage III, 2 patients (11.11%) in stage IV, 3 cases (16.67%) in stage V, and 4 cases (22.22%) in stage VI.

### SWE of thenar eminence

**Fig. 3:**
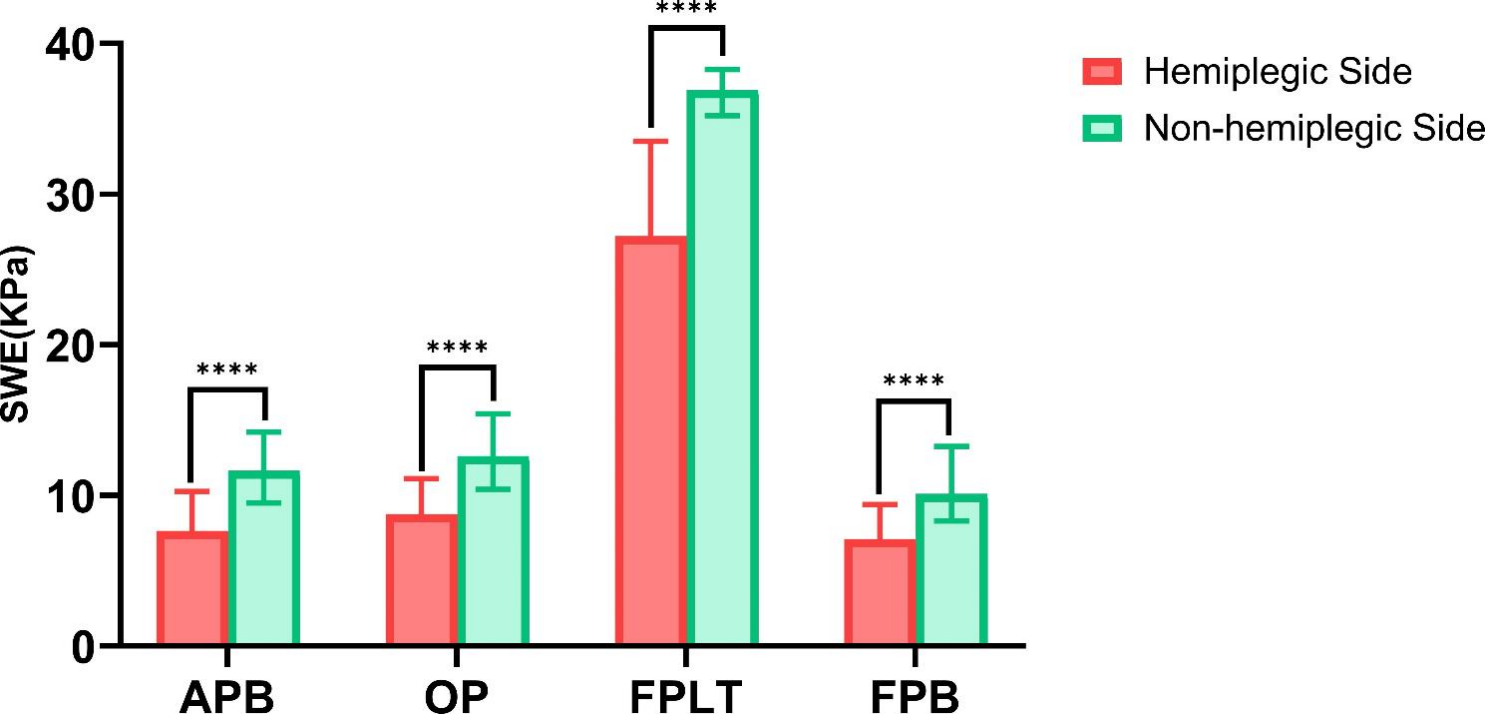
SWE Values of Thenar in Hemiplegia Patients **Notes**: There were statistical differences in the Mann-Whitney U test of SWE values of APB, FPB, OP and FPLT between the hemiplegic side and non-hemiplegic side of patients (P < 0.001).

The median SWE values of the hemiplegic side of patients were all lower than those of the non-hemiplegic side (Fig. 3, P < 0.001). By taking the SWE values of APB, FPB, OP, and FPLT of the major thenar of the hand on the non-hemiplegic side as the reference, SWE_R_ was calculated. APB-SWE_R_, FPB-SWE_R_, OP-SWE_R_ and FPLT-SWE_R_ were grouped according to the Bruunstrom stage, and there were statistical differences between groups (*P* < 0.001, Table 2).

**Table 2:** SWE_R_ of APB, OP, FPLT, FBP in different Bruunstrom stage

	Total	Bruunstrom Stage						P
		I	II	III	IV	V	VI	
APB	0.46 (0.07, 0.81)	0.56 (0.54, 0.68)	0.37 (0.32, 0.48)	-0.83 (-1.13, -0.50)	0.27 (0.18, 0.37)	0.18 (0.11, 0.25)	0.04 (0.01,0.10)	< 0.001
OP	0.44 (0.66, 1.01)	0.62 (0.49, 0.67)	0.40 (0.36, 0.46)	-1.16 (-1.69, -0.69)	0.28 (0.20, 0.32)	0.13 (0.10, 0.32)	0.46 (0.26,0.59)	< 0.001
FPLT	0.49 (0.07, 0.9)	0.46 (0.37, 0.50)	0.39 (0.33, 0.45)	-0.27 (-0.445, -0.16)	0.24 (0.13, 0.30)	0.17 (0.14, 0.20)	0.06 (0.01,0.09)	< 0.001
FPB	0.37 (0.08, 0.77)	0.50 (0.43, 0.66)	0.33 (0.27, 0.42)	-0.441 (-1.7, -0.27)	0.31 (0.14, 0.38)	0.16 (0.14, 0.25)	0.03 (0.0,0.07)	< 0.001

**Notes**: Variable reported as the median (inter-quartile range, IQR), Kruskal-Wallis test for comparison between six groups, the significance level is 0.05.

**Abbreviations**: APB, abductor pollicis brevis; OP, opponens pollicis; FPLT, flexor pollicis longs tendon, FPB, flexor pollicis brevis.

### Establishment of the Bayes discriminant function model

**Fig. 4:**
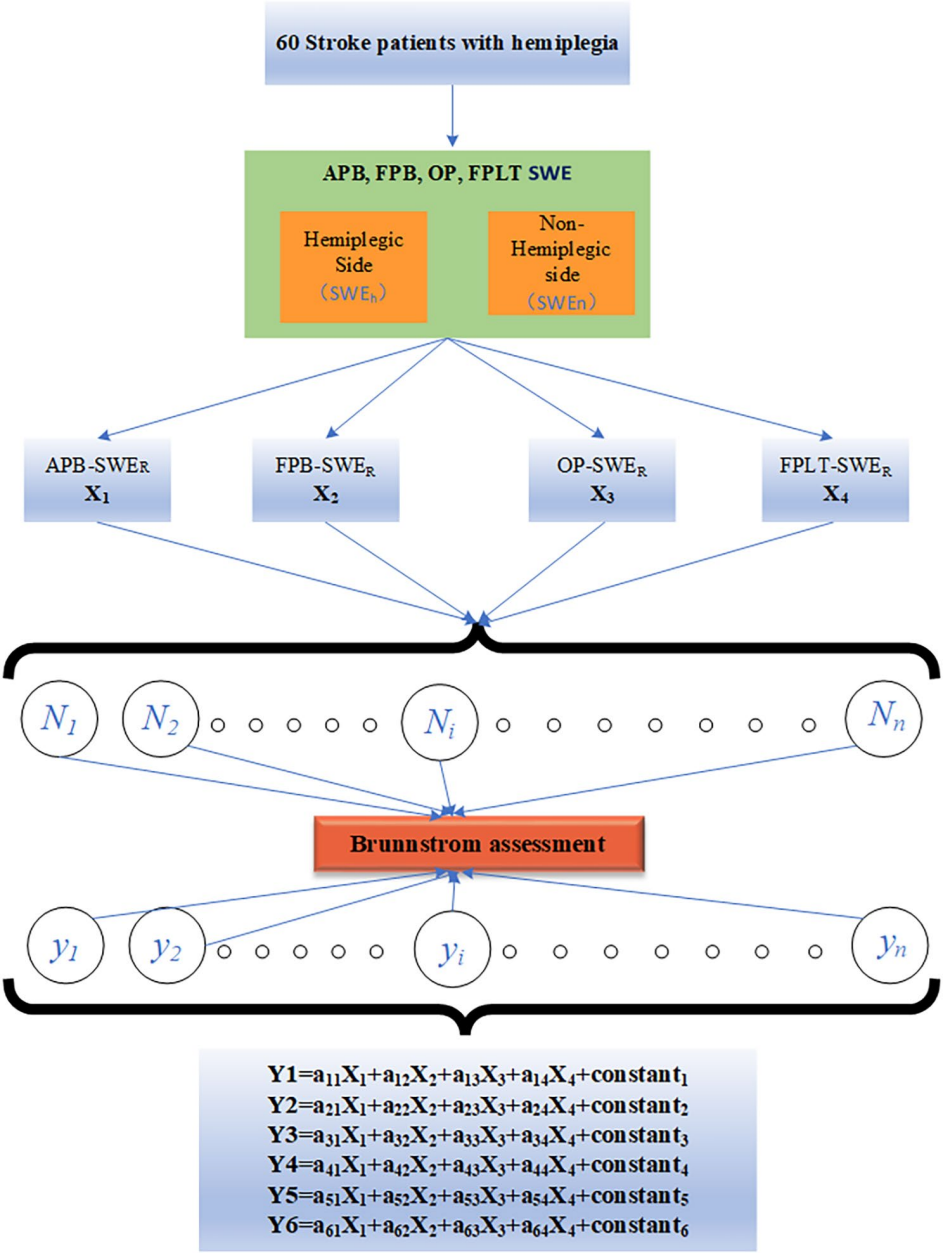
Path Diagram of Bayes Model Equation **Notes**: The meanings of a1…a6 in the route map are the same as coefficient results of discriminant function of in Table 3. X1, X2, X3, X4 in the map are the observation indexes of Y. Y indicates the stage of Brunnstrom assessment scale.

The results of this study implied that there were statistical differences among the six stages of rehabilitation in APB-SWE_R_, FPB-SWE_R_, OP-SWE_R_, and FPLT-SWE_R_ of patients (Table 2). For this reason, all of them were incorporated into the variables of the Bayes discriminant model. This study established a discriminant equation based on APB-SWE_R_, FPB- SWE_R_, OP-SWE_R_, FPLT-SWE_R_, and BA scale: Y = a1 × 1 + a2 × 2 + a3 × 3 + a4 × 4 + constant, where “a” was the coefficient, and X1 = APB-SWE_R_, X2 = FPB- SWE_R_, X3 = OP-SWE_R_, and X4 = FPLT-SWE_R_. In the first phase, 60 patients were included in this study. A Bayes discriminant model was set up, and six discriminant equations were obtained (Fig. 4). The specific discriminant coefficient is shown in Table 3 below. The independent variable data were substituted into a set of equations for the defect level of each cognition factor, and the maximum y value obtained was the rehabilitation stage corresponding to the research object.

**Table 3:** Coefficient results of discriminant function of Bruunstrom stage in hemiplegic

	Bruunstrom Stage					
	I	II	III	IV	V	VI
APB	-1.522	-1.237	4.340	-1.529	-0.914	0.122
OP	16.666	13.143	-37.077	7.821	4.661	1.250
FPLT	25.242	21.590	-28.709	12.571	10.601	2.749
FPB	-0.654	-1.638	7.485	0.701	0.434	-0.233
Constant	-11.858	-8.241	-25.508	-4.215	-3.187	-1.889

### Results and test of discriminant function

#### Self-validation and cross-validation method

To verify the accuracy of the discriminant equation, the independent variable data of the research object were put back into the equation using self-validation and compared with the BA stage of patients’ actual hand function. The Y-value determined the prediction stage of the hand function of each patient. The results showed that the correct recognition rate was 83.3%, and the misjudgment rate was 16.7% in the rehabilitation stage.

To evaluate the effect of the discriminant model, the discriminant function established for the above 60 patients was cross-checked by leave-one-out cross-validation, and the correct judgment rates were 73.3% and 26.7%.

#### External data validation method

According to the established Bayes equation, the hand function of 18 hospitalized hemiplegic patients was validated double-blindly. For 18 patients, the SWE values of APB FPB, OP, and FPLT on the hemiplegic and non-hemiplegic sides were measured, and APB-SWE_R_ FPB-SWE_R_, OP-SWE_R_, FPLT-SWE_R_ was calculated. The data were substituted into the diagnostic model to verify the diagnostic ability of the model. The correct recognition rate was 78.8%, and the misjudgment rate was about 21.2%.

#### Discriminant performance of the model

**Fig. 5:**
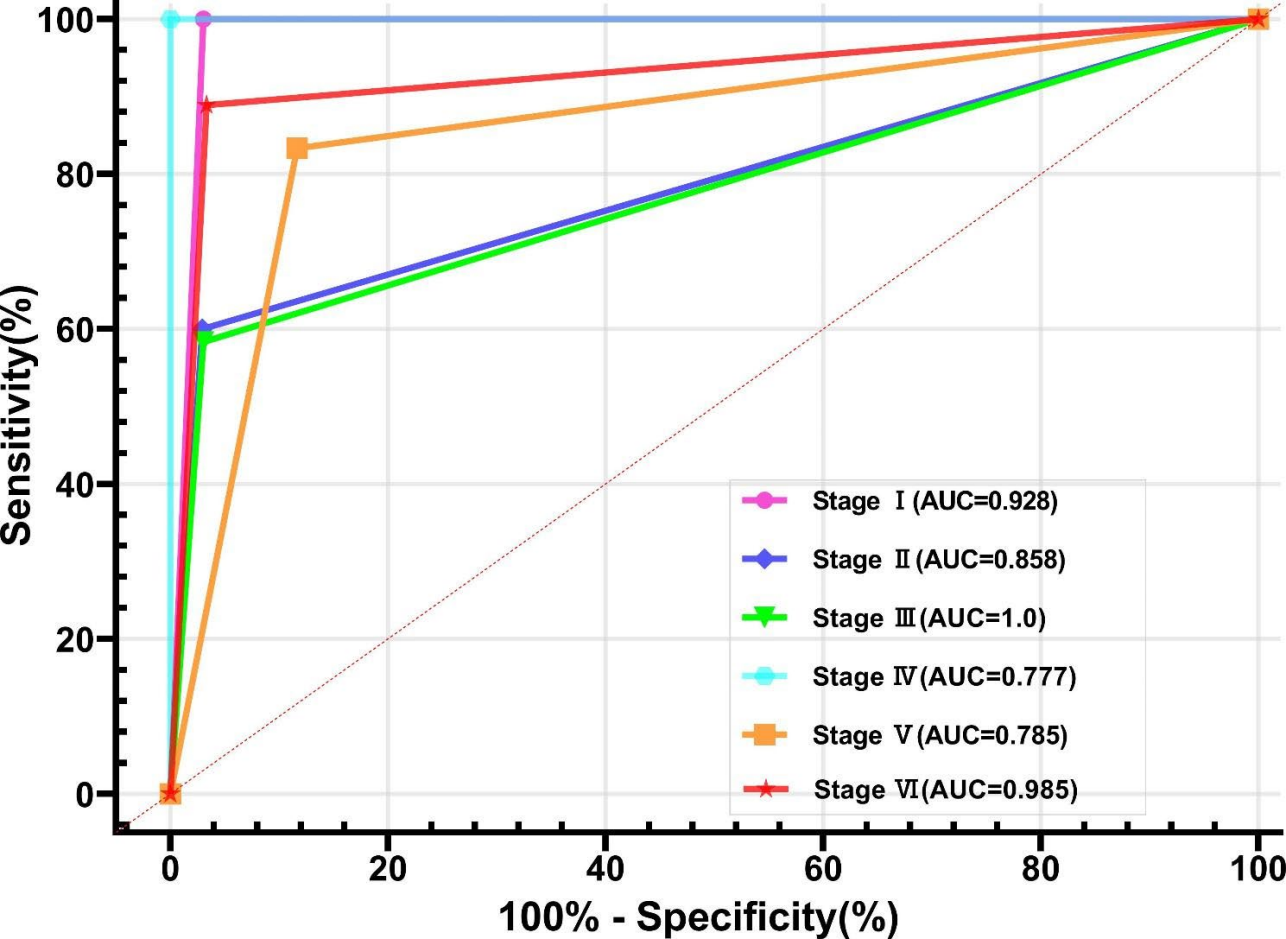
The diagnostic value of the Bayes discriminant function model with ROC curves **Notes**: Receiver operating characteristic (ROC) curves of Bayes discriminant function model for the BA stage of patients’ hand function.

The patients in the training group (n = 60) and the external test group (n = 18) after hemiplegia were used to test the diagnostic performance of the model. After the patient information is included in the Bayesian model, the patient hand function BA stage is recognized and automatically generated. The diagnostic performance is illustrated by the ROC curve (n = 78). The AUC of the discriminant model for diagnosing BA stage I-VI was 0.928(95% CI: 0.839-1.0),0.858(95% CI: 0.748–0.969),1.0(95% CI: 1.0–1.0), 0.777(95% CI: 0.599–0.954),0.785(95% CI: 0.593–0.977) and 0.985(95% CI: 0.959-1.0), respectively, with a sensitivity of 88.9%, 83.3%, 100%, 58.3%, 60% and 100% respectively, and a specificity of 96.7%, 88.3%, 100%, 96.7%, 97.1%, and 96.9% respectively (Fig. 5; Table 4).

**Table 4:** Discriminant performance of Bayes model for the BA stage of patients’ hand function

Bruunstrom Stage	AUC (95% CI)	Sensitivity (95% CI)	Specificity (95% CI)
I	0.928(0.839-1.0)	0.889(0.672–0.980)	0.967(0.886–0.994)
II	0.858(0.748–0.969)	0.833(0.608–0.942)	0.883(0.778–0.942)
III	1.0(1.0–1.0)	1.0(0.646-1.0)	1.0(0.949-1.0)
IV	0.777(0.599–0.954)	0.583(0.319–0.807)	0.967(0.896–0.995)
V	0.785(0.593–0.977)	0.60(0.313–0.832)	0.971(0.899–0.995)
VI	0.985(0.959-1.0)	1.0(0.75-1.0)	0.969(0.891–0.995)

## Discussion

During the early onset of stroke, more than 85% of patients had upper limb dysfunction, especially in the hands [10]. Major thenar directly got involved in the pinching and holding hands [11, 12], which was an important muscle group for completing hand function. The recovery of hand function after stroke is usually accompanied by changes in the characteristics of muscle tissues, including the shortening of the sarcomere, accumulation of connective tissue, changes in muscle stiffness, etc. [13, 14]. They change dynamically with the progression of the disease. The changes in muscle stiffness can reflect changes in the rehabilitation stage. However, the clinical judgment of rehabilitation degree mainly depends on the subjective assessment of therapists, without quantitative analysis evidence. SWE can be used to quantify muscle characteristics and has been applied in studies evaluating muscle stiffness in neuromuscular diseases [15–17]. Young’s modulus derived from SWE measurement is a physical quantity to describe the resistance of a solid matter to deformation and is directly proportional to the stiffness of matter. SWE can be used to observe changes in muscle stiffness, including reflex stiffness caused by increased excitability of motor neurons and increased muscle stiffness due to changes in mechanical properties of muscles. Discriminant analysis is widely applied in many medical fields [18–20].

This study aimed to analyze quantitatively, establish a discrimination model for rehabilitation stages of hand function and provide a automatic and quantitative basis for clinical evaluation by measuring the thenar SWE of hemiplegic patients after stroke. Previous studies found no significant difference from healthy individuals in the stiffness of bilateral skeletal muscles [8]. Thus, by taking the non-hemiplegic side as the reference, this study calculated the change rate of SWE to minimize the effect of individual differences in muscle stiffness on analysis. Through a comprehensive evaluation of APB-SWE_R_, FPB- SWE_R_, OP-SWE_R_, and FPLT-SWE_R_, the rehabilitation degree was evaluated from the changes in muscle stiffness, and a Bayes discriminant model was established to intuitively distinguish and predict the rehabilitation degree in the form of data.

The results of the discriminant function suggested that the correct judgment rate of self-validation was 83.3%, and the right judgment rate of cross-validation was 73.3%. Since self-validation and cross-validation often underestimated the misjudgment rate and exaggerated the judgment effect, we estimated the misjudgment probability prospectively for the diagnostic model using validation samples. The misjudgment probability obtained with this method was objective. A total of 14 patients were correctly judged, and 4 patients were wrongly judged, with a correct rate of 78.8%. In addition, AUC also indicates the diagnostic value of using SWE_R_ to create Bayes discriminant model in diagnosing various rehabilitation stages.

### Limitations

As an evaluation model applied to clinical practice, despite the small sample size of this study, this is probably why the correct rates of cross-validation and external validation are lower than that of self-validation. In the future, with respect to the validation of the hand function evaluation model, we still need to expand the sample size and establish a decision function with higher accuracy and better stability, to provide more direct and reliable evaluation indicators for clinical rehabilitation effect and outcome of hand function.

## Conclusion

The validation of external data, cross-validation, and evaluation of classification performance through the area under the ROC curve (AUC), sensitivity, and specificity showed that the established Bayes discriminant model had small fluctuations and acceptable stability, which can be applied to a great many sample studies, eliminating the disadvantages of evaluation consistency in clinical work and significant fluctuations in evaluation results, save time and energy, and has great reference value for clinical diagnosis, treatment, and outcome of rehabilitation degree.

## Data Availability

The data supporting the findings of this study are available upon request from the corresponding author.
